# The role of pioglitazone in antioxidant, anti-inflammatory, and insulin sensitivity in a high fat-carbohydrate diet-induced rat model of insulin resistance

**DOI:** 10.1590/1414-431X2020e10782

**Published:** 2021-05-24

**Authors:** H.M. Al-Muzafar, F.S. Alshehri, K.A. Amin

**Affiliations:** 1 Department of Chemistry, College of Science, Imam Abdulrahman Bin Faisal University, Dammam, Saudi Arabia; 2 Basic & Applied Scientific Research Center, Imam Abdulrahman Bin Faisal University, Dammam, Saudi Arabia

**Keywords:** Pioglitazone, Type 2 diabetes mellitus, Antioxidants, Insulin resistance, Anti-inflammatory

## Abstract

We explored the cascade effects of a high fat-carbohydrate diet (HFCD) and pioglitazone (an anti-diabetic therapy used to treat type 2 diabetes mellitus (T2DM)) on lipid profiles, oxidative stress/antioxidant, insulin, and inflammatory biomarkers in a rat model of insulin resistance. Sixty albino rats (80-90 g) were randomly divided into three dietary groups; 1) standard diet; 2) HFCD diet for 12 weeks to induce an *in vivo* model of insulin resistance; and 3) HFCD diet plus pioglitazone. Blood and tissue samples were taken to assess hepatic function, lipid profiles, oxidative biomarkers, malondialdehyde (MDA) levels, antioxidant defense biomarkers, including reduced glutathione (GSH), superoxide dismutase (SOD), and the inflammatory markers interleukin-6 (IL-6) and tumor necrotic factor (TNF-α). HFCD-fed rats had significantly (P≤0.05) increased serum triacylglycerol (TG), total cholesterol (TC), low-density lipoprotein (LDL), alanine transaminase (ALT), and bilirubin levels, but decreased high-density lipoprotein (HDL) levels compared with the normal group. Moreover, serum leptin, resistin, TNF-α, and IL-6 levels were increased significantly in HFCD animals compared with controls. Similarly, HFCD-induced insulin resistance caused antioxidant and cytokine disturbances, which are important therapy targets for pioglitazone. Importantly, administration of this drug ameliorated these changes, normalized leptin and resistin and inflammatory markers by reducing TNF-α levels. Metabolic cascades of elevated lipid profiles, oxidative stress, insulin, and inflammatory biomarkers are implicated in insulin resistance progression. HFCD induced metabolic cascades comprising hypertriglyceridemia, hyperglycemia, insulin resistance, obesity-associated hormones, and inflammatory biomarkers may be alleviated using pioglitazone.

## Introduction

Excess dietary fat and carbohydrate disturbances are the main causes of metabolic disorders, especially hepatic steatosis, metabolic syndrome, type 2 diabetes mellitus (T2DM), and obesity (1). Diabetes mellitus (DM) is a collection of metabolic syndromes represented by hyperglycemia, inadequate insulin synthesis, and improper responses to insulin (2). Approximately 85-90% of diabetics have T2DM characterized by insulin resistance, meaning circulating insulin cannot bind to receptors thereby limiting downstream biochemical functions, i.e., glycolysis, glycogenesis, lipogenesis, and protein anabolism. Mechanistically, insulin resistance is potentially linked to inflammatory response (3) and oxidative stress.

Insulin resistance in muscle and liver is influenced by large fat deposits in extended adipose tissue bulk, thereby contributing to obesity (4). Globally, obesity has increased dramatically, with approximately 300 million people affected. The condition is also a substantial risk factor for T2DM (5). Adipose tissue is the main energy source in the body, where resident macrophages secrete numerous proteins (6).

A high fat-carbohydrate diet (HFCD) potentially induces non-alcoholic fatty liver disease (NAFLD), which is characterized by fat accumulation in the liver and is manifested as mild, moderate, and severe hepatosteatosis (7,8). Mechanistically, this is the most widely accepted model for insulin resistance, when compared with other models. Thus, insulin resistance is associated with elevated liver fatty acids, peripheral lipolysis, and triacylglycerol (TG) synthesis, which stimulate hepatic lipid accumulation (7,8).

High fat diet (HFD)-induced obesity initiates inflammatory responses and insulin resistance via several mechanisms (9). Understanding these pathways is vital if novel therapies are to treat diabetes, metabolic syndrome, and its complications (NAFLD and cardiovascular disorders). One such mechanism involves lipid accumulation that potentially leads to adipocyte demise and increased intestinal lipopolysaccharide (LPS) leakage, thereby activating tissue inflammatory reactions (9,10). Similarly, free fatty acids (FFAs) from HFD and LPS stimulation of toll-like receptors increase cytokine production and ultimately lead to immune cell aggregation (11).

Macrophages in fatty tissues are the main cause of inflammatory marker production, i.e., interleukin-6 (IL-6) and tumor necrotic factor (TNFα) (11). During normal homeostasis, these adipokines regulate energy metabolism and maintain both energy expenditure and intake, in addition to insulin sensitivity (5). However, during disease pathology, e.g., diabetes and insulin resistance, chronic low-grade inflammation is generated by these molecules. Furthermore, obesity is linked to inflammation in white adipose tissue (WAT), culminating in glucose intolerance, insulin resistance, and diabetes (12).

Another vital element of HFCD-induced fatty tissue inflammation and insulin resistance is macrophage permeation. Macrophage migration inhibitory factor (MIF) is a potential therapeutic for HFD-induced insulin resistance and controlling its activities with pharmacological mediators or functional foods could be therapeutically viable for the treatment and prevention of obesity-related metabolic disorders and insulin resistance (13).

The transcription factor, peroxisome proliferator-activated receptor gamma (PPARγ), regulates lipogenesis, adipokine discharge, and hormones implicated in carbohydrate and lipid metabolism (14). Thiazolidinediones (glitazones) are a family of PPARγ ligands and are potent oral insulin sensitizers used for T2DM therapy. The insulin-sensitizing therapies, pioglitazone and rosiglitazone, are PPAR-γ agonists with hypoglycemic roles. They modify adipose tissue functions (15) and decrease inflammatory reactions in T2DM patients and poly-microbial sepsis by inhibiting NF-κB and are now a new treatment for sepsis (16).

Lipid and inflammatory dysfunction, oxidative stress, and insulin resistance are key T2DM treatment areas. Several less well-established therapeutic drugs have been developed as effective therapies for T2DM. As oxidative stress is a significant issue in insulin resistance and T2DM, most therapeutic approaches avoid reactive oxygen species (ROS) overproduction and inflammatory responses (17).

Therefore, we explored lipid profiles, oxidative stress, inflammatory biomarkers, and insulin resistance cascades, and their potential applications as biomarkers and therapeutic targets using pioglitazone. We also assessed the impact of pioglitazone on cytokine levels, insulin, and inflammatory mechanism in HFCD-induced insulin resistance.

## Material and Methods

### Animal diet

Two rat food types were used: a regular rat chow and HFCD (PE Enterprise, Kingdom of Saudi Arabia). The standard rat chow comprised 5% fat, 65% carbohydrates, 20% crude protein, composed of 350 g of soya bean, whey protein, and meat, 5% vitamins and minerals, 50 g dicalcium phosphate, calcium carbonate, magnesium oxide, and sodium chloride, and 5% dietary fiber. The amount of energy of this diet was 2813 kcal/kg.

The HFCD comprised 20% fat (200 g saturated fatty acid/kg), 55% carbohydrates, 20% crude protein, and 5% vitamins and minerals (18). The amount of energy of this diet was 5100 kcal/kg.

### Study animals

Sixty 6-week-old male Albino rats with an average weight of 80-90 g were provided by King Abdulaziz City for Science and Technology (KACST), Saudi Arabia. Rats were maintained under observation for one week after their arrival to the laboratory. All rats were separated in plastic pens at 24±3°C, with 12-h light/dark cycles at 40-60% moisture in the laboratories of Institute for Research and Medical Consultations (IRMC), Imam Abdulrahman Bin Faisal University. Rats had *ad libitum* access to food and water.

The animal experiments were carried out in accordance with the National Institutes of Health guidelines for the care and use of laboratory animals (NIH, USA). We certify that this work was performed in accordance with local ethical guidelines, with the approval of Imam Abdulrahman University's Institutional Review Board (IRB; number IRB-21049066).

### Study design and animal grouping

The study lasted 16 weeks and was conducted over two phases: 1) insulin resistance induction model (weeks 1-12); and 2) therapy (weeks 12-16). Sixty rats were randomized to three groups, each comprising 20 rats. Twenty animals in the normal control group were fed a normal rat chow for the study duration (16 weeks). In the HFCD group, 40 rats were fed HFCD. From week 12-16, the HFCD group (n=40) was split into two groups. The positive control group (n=20) remained on the HFCD diet, whereas the other group received a daily pioglitazone dose at 17.5 mg/kg body weight orally for four weeks in a volume equivalent to 5 mL/kg.

### Blood analysis

Blood samples were assayed for total cholesterol (TC), triacylglycerol (TGs), high-density lipoprotein (HDL), low-density lipoprotein (LDL), and bilirubin levels. These were calorimetrically assayed using kits from Human Gesellschaft fur Biochemica und Diagnostica mbH (Germany). TGs were enzymatically measured in serum through TG hydrolysis by lipase to produce glycerol. The glycerol was then oxidized using glycerol oxidase and H_2_O_2_, leading to the formation of a quinoneimine dye that was measured, as described below, for cholesterol measurement. Absorbance was measured at 520 nm. Cholesterol measurements depend on the hydrolysis of cholesterol esters in producing cholesterol that can be oxidized. The dye color intensity is proportional to cholesterol concentration. In a coupled reaction catalyzed by peroxidase, red quinoneimine dye was formed from 4-aminoantipyrine+p-HBS+H_2_O_2_ with absorbance at 520 nm.

The principle of HDL determination was quantitatively applied in serum that reacted with the polyethylene glycol reagent, all the LDL and very low-density lipoprotein (VLDL) were precipitated, and the HDL fraction remained in the supernatant. The supernatant was then treated as a sample for the cholesterol assay. Friedewald formula cannot be applied to estimate the LDL-cholesterol in rodent serum samples. The direct LDL reagent kits (TECO Diagnostics, USA) comprised a two-part, liquid stable method for directly measuring LDL-C levels in serum. This method depends on the properties of a unique detergent to remove any pre-treatment or centrifugation steps. The detergent (Reagent 1) solubilizes only the non-LDL particles. The cholesterol released is consumed by the cholesterol esterase and cholesterol oxidase in a non-color forming reaction. A second detergent (Reagent 2) solubilizes the remaining LDL particles, and a chromogenic coupler allows for enzymatic color formation that is proportional to the amount of LDL present in the sample.

The glucose assay is based on glucose oxidation by glucose oxidase into D-gluconic acid plus hydrogen peroxide, which was detected with a specific colorimetric probe. Horseradish peroxidase catalyzes the reaction between the probe and hydrogen peroxide. Samples were compared to a standard glucose. Samples and standards were incubated for 30-45 min and measured with a colorimetric plate reader (BEPIII system-ELISA, Siemens Co., USA).

Lipid peroxidation (MDA) colorimetric assay kit (BioVision Inc., USA) was used to quantify oxidative stress biomarkers. Lipid peroxidation forms malondialdehyde (MDA) as natural biproducts, where the sample MDA reacts with thiobarbituric acid (TBA) to generate the MDA-TBA adduct that is easily measured colorimetrically at 532 nm absorbance. This assay detects MDA levels as low as 1 nmol/well.

BioVision's ApoGSH^TM^ glutathione colorimetric assay kit was used to measure glutathione (GSH). The assay is based on the glutathione recycling system by DTNB and glutathione reductase. DTNB and GSH react to generate 2-nitro-5-thiobenzoic acid, which has yellow color. Therefore, GSH concentration can be determined by measuring absorbance at 412 nm.

Superoxide dismutase (SOD) activity assay kit was obtained from BioVision Inc. The assay kit utilizes WST-1 that produces a water-soluble formazan dye upon reduction with a superoxide anion. The rate of the reduction with a superoxide anion is linearly related to the xanthine oxidase activity and is inhibited by SOD. Therefore, the inhibition activity of SOD can be determined by a colorimetric method.

### ELISA and immunoassays

Leptin and resistin levels were quantified by enzyme-linked immunosorbent assay (ELISA) from SPI Bio-Product PTY Ltd. (France). The principle of leptin is that it is an enzymatically amplified, two-step, sandwich-type immunoassay, where standards, controls, and unknown serum samples are incubated in micro-titration wells coated with anti-leptin antibody. After a two-step incubation and washing process, stopping solution is added, and the degree of enzymatic turnover of the substrate is measured by chromatography. Resistin ELISA kit is intended for the quantitative measurement of resistin protein in serum. ELISA utilizes the complex immunocapture antibody/sample analyte/conjugated detector antibody generating blue coloration that stopped the solution from completing any color change from blue to yellow. The signal is generated proportionally to the amount of bound analyte and the intensity is measured at 450 nm.

TNF-α and IL-6 ELISA kits were purchased from Abcam (USA). TNF-α or IL-6 ELISA employs an antibody specific for TNFα or IL-6 coated on a well plate and bound to TNF-α or IL-6 present in the standards and samples. The wells are washed, biotinylated anti-IL-6 or anti-IL-6 antibody, HRP-conjugated streptavidin is added, washed again, followed by a TMB substrate solution, and color develops in proportion to the amount of TNF-α or IL-6 bound. The stop solution changes the color from blue to yellow, and the intensity of the color is measured at 450 nm in a microplate reader, and the concentrations of TNFα and IL-6 are calculated.

Serum insulin was measured using insulin rat enzyme immunoassay kits (Berti Pharma, France). Insulin EIA kits depend on competition between unlabeled rat insulin and acetylcholinesterase linked to rat insulin for limited specific Guinea-pig anti-rat insulin antiserum sites that bind to goat anti-Guinea-pig antibody that attached to the wells. The plate is washed and Elman's reagent is added to form a yellow compound where its intensity is determined spectrophotometrically.

### Study drug

Pioglitazone hydrogen chloride was purchased from Santa Cruz Biotechnology Inc. (USA). The molecular formula is C_19_H_21_ClN_2_O_3_S and the molecular weight is 392.9 g/mol. In total, 70 mg pioglitazone powder was dissolved in 20 mL distilled sterile water. Each rat received an oral administration (*os*) dose of 1 mL per day at 17.5 mg/kg body weight for four weeks (14,15).

### Blood and tissue sampling

Blood samples were harvested using micro-hematocrit capillary tubes from the medial canthus of the eye during fasting. To generate serum, blood was collected in dry centrifuge tubes and clotted at 24±3°C before centrifugation at 1400 *g* for 20 min at 20^o^C. The clear, non-hemolyzed serum was aspirated by syringe and stored at -80°C for downstream analysis. At study end, all rats were sacrificed by anesthesia, and liver slices were fixed in 10% formalin for histopathology.

### Blood biochemistry and histopathology

Serum was used to assay lipid profiles, comprising TG, TC, LDL, and HDL. Equally, hepatocellular integrity was investigated by measuring alanine transaminase (ALT), bilirubin, and albumin levels. Histopathology was performed on tissues using hematoxylin and eosin (H&E) staining. Microscopic analysis assessed hepatocyte morphology, the presence/lack of fat globules among hepatocytes and inflammatory cells, and gross hepatocyte pathology.

### Insulin determination and calculation of HOMA-IR index

Fasting serum insulin was measured by ELISA (Biorbyt, UK) at 450 nm following manufacturer's instructions. The homeostasis model assessment-insulin resistance (HOMA-IR) index was conducted according to Matthews et al. (19): HOMA IR = serum insulin (mM) × blood glucose (mM) / 22.5.

### Statistical analysis

Data were statistically evaluated using one-way analysis of variance (ANOVA), followed by the Tukey-Kramer method for *post hoc* analysis. Data are reported as means±SE, and values were considered statistically significant when P<0.05. Statistical analyses were performed using GraphPad Prism 6 software (USA).

## Results

Hepatocellular damage markers (ALT) and direct bilirubin levels were higher in HFCD-fed rats compared with controls. We also observed that pioglitazone treatment decreased enzyme activities (ALT) compared with controls (Table 1).

**Table 1:** Effect of high fat-carbohydrate diet (HFCD) and HFCD+pioglitazone on liver integrity, alanine transaminase (ALT), total proteins, total bilirubin, and direct bilirubin.

	Normal	HFCD	HFCD + pioglitazone
ALT (U/L)	30.8±1.94	49.3±4.39***	37.2±0.90
Total proteins (g/dL)	8.41±0.69	8.41±0.56	8.11±0.55
Total bilirubin (mg/dL)	1.42±0.16	2.36±0.23**	1.73±0.17
Direct bilirubin (mg/dL)	0.79±0.09	2.08±0.1***	1.51±0.13

Data are reported as means±SE. **P<0.01, ***P<0.001 compared to HFCD+pioglitazone (ANOVA).

Rats on HFCD generated higher blood glucose, insulin, and HOMA-IR indices compared with controls. In contrast, pioglitazone therapy significantly reduced these indices compared with the HFCD group (P<0.05, Table 2).

**Table 2 t02:** Effect of high fat-carbohydrate diet (HFCD) and HFCD+pioglitazone on glucose, insulin, and homeostasis model assessment-insulin resistance (HOMA-IR) index.

	Normal	HFCD	HFCD + pioglitazone
Glucose (gm/dL)	114.64±2.93^a^	189.44±17.25^b^	137.51±4.05^c^
Insulin (ng/mL)	3.44±0.32	5.3±0.52	4.86±0.22
HOMA-IR	0.56±0.03^a^	0.98±0.11^b^	0.73±0.043^a^

Data are reported as means±SE. Different superscript letters indicate a significant difference at P≤0.05 (ANOVA).

Oxidative/antioxidant biomarkers, as evaluated by MDA and GSH levels, indicated alterations among experimental groups. Statistical analyses indicated significantly higher MDA and lower GSH levels in the HFCD group compared with normal control, however, pioglitazone therapy decreased MDA and increased GSH levels (Table 3). Table 4 shows the correlation coefficient between markers, signifying correlations between TG and insulin levels.

**Table 3:** Effect of high fat-carbohydrate diet (HFCD) and HFCD+pioglitazone on oxidative stress biomarker malondialdehyde (MDA) and antioxidant biomarkers reduced glutathione (GSH) and superoxide dismutase (SOD) in the blood.

Oxidative/antioxidant markers	Normal	HFCD	HFCD + pioglitazone
MDA (nmol/mL)	1.56±0.17	2.23±0.08	1.31±0.25
GSH (ng/μL)	1.39±0.15^a^	0.773±0.12^b^	1.42±0.21^a^
SOD (U/mL)	87.7±3.38	157±19.5	94.4±1.76

Data are reported as means±SE. Different superscript letters indicate a significant difference at P≤0.05 (ANOVA).

**Table 4:** Correlation coefficient between biomarkers.

	TG	Cholesterol	LDL	Glucose	Insulin
TG					
Pearson Correlation	1	0.047	-0.219	0.120	-0.595^*^
Significance (2-tailed)		0.867	0.415	0.682	0.019
N	17	15	16	14	15
Cholesterol					
Pearson Correlation	0.047	1	0.070	-0.197	-0.025
Significance (2-tailed)	0.867		0.805	0.501	0.931
N	15	15	15	14	15
LDL					
Pearson Correlation	-0.219	0.070	1	0.011	0.288
Significance (2-tailed)	0.415	0.805		0.970	0.297
N	16	15	16	14	15
Glucose					
Pearson Correlation	0.120	-0.197	0.011	1	-0.291
Significance (2-tailed)	0.682	0.501	0.970		0.313
N	14	14	14	14	14
Insulin					
Pearson Correlation	-0.595^*^	-0.025	0.288	-0.291	1
Significance (2-tailed)	0.019	0.931	0.297	0.313	
N	15	15	15	14	15

Correlation is significant at the 0.05 level (2-tailed). TG: triacylglycerol; LDL: low-density lipoprotein.

**Figure 1:**
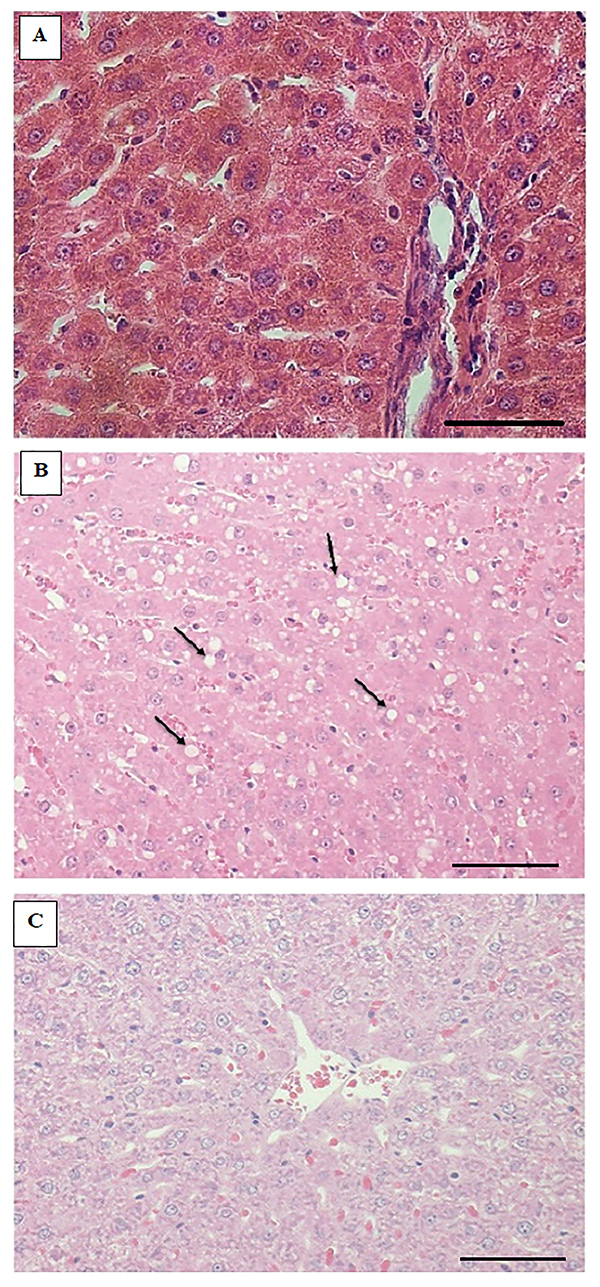
Histopathological changes in normal, high fat-carbohydrate diet (HFCD) and HFCD+pioglitazone groups. **A**, Normal hepatocytes without inflammatory cells. **B**, Microscopic photo of the liver presented numerous fat droplet (arrows) among and within the liver cells, with associated deteriorating changes in hepatocytes in the HFCD group. **C**, Normal hepatocytes without inflammatory cells and low fat globules in the HFCD+pioglitazone group. H&E stain, magnification 20×, scale bar 200 μm).

**Figure 2:**
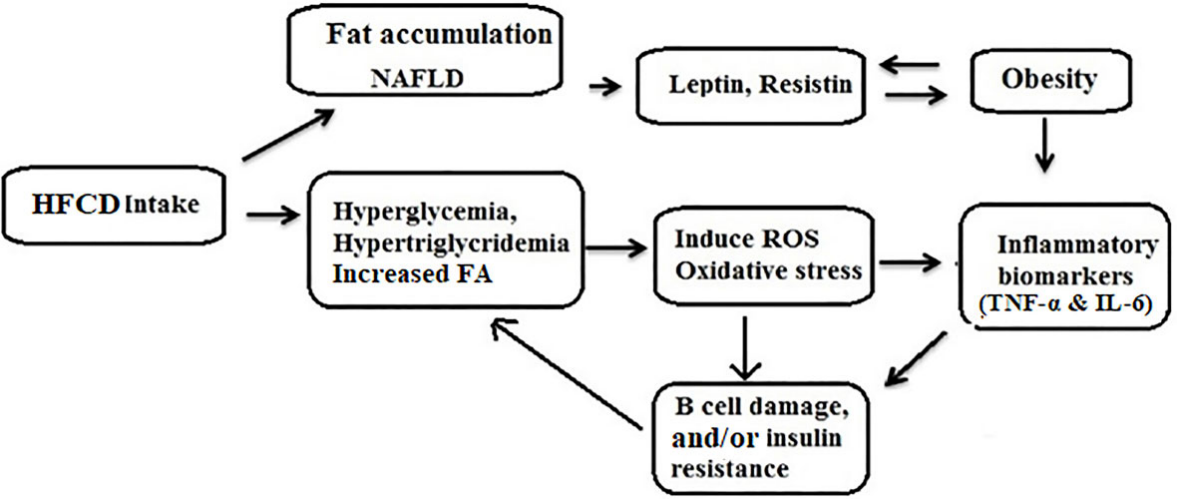
Mechanism of action of the high fat-carbohydrate diet (HFCD), oxidative stress, insulin resistance, and inflammatory markers. Mechanism of action of the different treatment groups: Biochemical cascade and mechanism of HFCD intake on the induction of reactive oxygen species (ROS) generation and inflammatory biomarkers (TNF-α, IL-6, obesity, free fatty acids (FFA), leptin, and insulin resistance). Interference with insulin resistance leads to hyperglycemia and inflammatory biomarkers changes (increased TNF-α and IL-6) lead to the inhibition of insulin signaling and insulin resistance. Also, inflammation in B cells leads to B cell dysfunction, which in combination with insulin resistance, results in type 2 diabetes mellitus. NAFLD: non-alcoholic fatty liver disease.

We also investigated histopathological changes of the different animal groups (Figure 1). In normal hepatocytes, we observed no inflammatory cells (Figure 1A). In hepatocytes/liver samples from the HFCD group, numerous fat droplets in and around liver cells were observed (Figure 1B). In the HFCD + pioglitazone group, normally shaped hepatocytes, without inflammatory cells were visualized with fewer fat globules (Figure 1C). The mechanism of action of the different treatment groups is shown in Figure 2.

**Figure 3:**
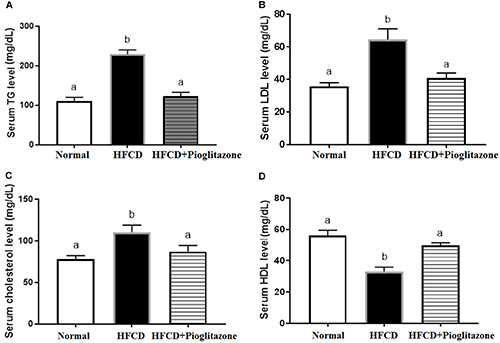
Changes in lipid biomarkers: triacylglycerol (TG), low-density lipoprotein (LDL), total cholesterol, and high-density lipoprotein (HDL) of normal, high fat-carbohydrate diet (HFCD), and HFCD+pioglitazone groups. Data are reported as mean±SE. Different letters indicate a statistical difference among groups (P<0.05, ANOVA).

Thus, rats on a 12-week HFCD experienced alterations in blood lipid profiles, with increases in TC, TG, and LDL levels, and a decrease in HDL levels compared with control animals. Pioglitazone administration decreased serum TC, TG, and LDL levels, whereas HDL levels were elevated compared with the HFCD group (Figure 3).

**Figure 4 f04:**
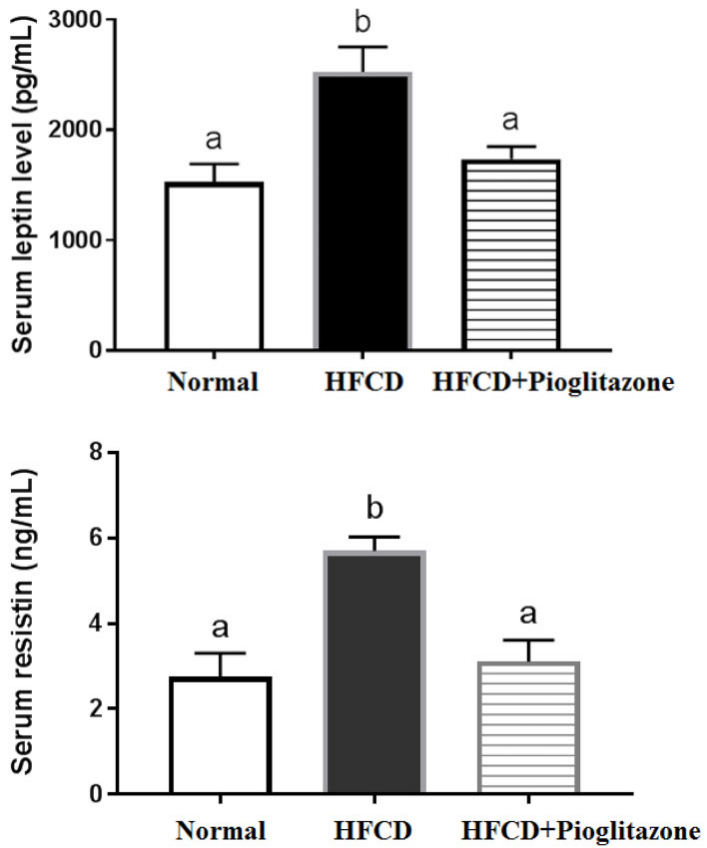
Changes in lipid hormones leptin and resistin of normal, high fat-carbohydrate diet (HFCD), and HFCD+pioglitazone. Data are reported as mean±SE. Different letters indicate statistical a difference among groups (P<0.05, ANOVA).

**Figure 5:**
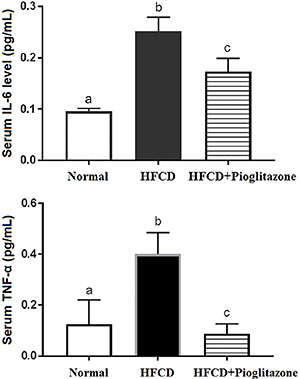
Role of high fat-carbohydrate diet (HFCD) and HFCD+pioglitazone on inflammatory markers interleukin-6 (IL-6) and tumor necrotic factor (TNF-α). Data are reported as mean±SE. Different letters indicate a statistical difference among groups (P<0.05, ANOVA).

The HFCD group exhibited increased leptin and resistin levels, while pioglitazone decreased these changes (Figure 4). For inflammatory markers, HFCD animals exhibited increased TNF-α and IL-6 levels compared to the control group. Similarly, pioglitazone therapy down-regulated these levels compared with HFCD animals (Figure 5). The data in Tables 1-3 and Figures 1,3-5 confirmed that a NAFLD was initiated, an insulin resistance rat model was established, and pioglitazone altered these changes to various extents.

## Discussion

Our data indicated associations between insulin resistance and NAFLD, with evidence suggesting that hepatic lipid accumulation, insulin resistance, oxidative stress, and inflammatory markers were involved in this association. We hypothesized that a cascade of excess carbohydrates and fats, lifestyle choices, and steatosis, accompanied by oxidative stress and inflammatory events, contribute to insulin resistance and subsequent T2DM and steatohepatitis. Therefore, rapid treatment regimens are required to break these pathological cascades.

The global incidence of obesity and DM is related to NAFLD (20), which is a reason for chronic hepatic disease and prevalence in approximately 30% of western nations. NAFLD could be considered as the hepatic indicator of insulin resistance and is intensely accompanied by the metabolic syndrome (21).

Our study indicated that HFCD-induced diabetes was associated with dyslipidemia, i.e., hypertriglyceridemia, decreased HDL, and increased LDL levels (Figure 3). These conditions are associated with disturbances in liver integrity and increased fatty deposition due to hepatosteatosis. Also, increased serum bilirubin may be due to hepatic tissue deterioration from fat accumulation and conjugate bilirubin with glucuronic acid passing to blood and increasing its level.

Moreover, the HFCD group showed elevated hyperglycemia and MDA levels compared with controls. Hyperglycemia and hypertriglyceridemia are severe diabetic risks linked to elevated diabetic complications associated with oxidative stress and inflammatory processes (22,23). Significant differences in glucose, TG, HDL, and TC between the control and HFCD groups supported the association of these markers with insulin resistance and demonstrated the existence of a continuum between increasing hyperglycemia and hypertriglyceridemia, and insulin resistance development.

Our data also indicated that pioglitazone improved lipid profiles, liver integrity (ALT), blood oxidative stress markers, and inflammatory biomarkers in our *in vivo* insulin resistance rat model. Pioglitazone exerted antioxidant and anti-inflammatory benefits towards insulin resistance and reduced hypertriglyceridemia and hyperglycemia compared to the HFCD group. These findings suggested pioglitazone was an effective insulin resistance therapy, when associated with NAFLD.

Global increases in obesity and insulin resistance are related to increases in NAFLD and T2DM (24). Both conditions are characterized by excess TG deposition in hepatic cells from an increased influx of FFAs and/or *de novo* lipid synthesis. Thus, these disease manifestations exert enormous clinical and economic burdens on society (25).

Insulin, HOMA-IR, and TG indices were increased in HFCD-induced insulin resistance animals (Tables 1 and 4 and Figure 3) compared with controls. Mechanistically, hepatosteatosis is linked to T2DM and insulin resistance via lipid accumulation in hepatocytes. This increases mitochondrial β-oxidation and substrate transport to mitochondrial respiratory chains, thereby elevating ROS production and ultimately respiratory chain dysfunction. Such exacerbations increase FA β-oxidation rates, culminating in the accumulation of incomplete metabolites. Elevated ROS as represented by MDA levels contributes to insulin resistance and T2DM progression (20,26). ROS and redox-sensitive metabolites are implicated in several signaling pathways, transcription factors, and nuclear receptors implicated in hepatic lipid metabolism (27).

Current T2DM pioglitazone therapies mediate oxidative stress, inflammatory markers, and insulin resistance risk factors (28). Stimulus of the mitochondrial function may avoid T2DM progress, protecting the cell from the high delivery of reduced coenzymes from glycolysis and lipid oxidation to the electron transport chain and production of ROS (29).

The cascade of high carbohydrate and fat accumulation increases β-oxidation, elevates ROS, promotes mitochondrial disturbances and pro-inflammatory cytokine release, leading to insulin resistance and T2DM (30). This pathophysiology is aggravated by islet of Langerhans (insulin secretors) dysfunction at the pancreas, exacerbating serum glucose concentrations.

An *in vivo* model system of insulin resistance is vital for the study of T2DM (non-insulin dependent diabetes mellitus, NIDDM). Most patients with T2DM are obese, and obesity causes a degree of insulin resistance. This form of diabetes often goes undiagnosed because hyperglycemia develops gradually and, at earlier stages, it is not severe enough to produce any of the classic symptoms of diabetes. This form of diabetes may have insulin levels that appear normal or increased that cause the inability of insulin to control hyperglycemia and lipid metabolism, and even β-cell function may be normal. In our study, HFCD induced insulin resistance via blocked insulin receptors, reduced glucose transport activity, and impaired glucose metabolism. Furthermore, hyper-caloric foods are known to promote cholecystokinin release triggering pancreatic expansion producing hyperinsulinemia, glucose intolerance, and insulin resistance, Therefore, insulin secretion is defective in T2DM and insufficient to compensate for insulin resistance and treatment is insulin-independent.

This information raised the investigation of the biochemical and molecular basis of the disease and novel strategies for its prevention and therapy. At the cellular level, HFCD increased FFA instability, and flux into the liver initiated a signaling cascade sequence that led to phosphorylation of substrates and cellular defects in the insulin signaling pathways.

In summary, HFCD could induce hepatosteatosis, the key for FA oxidation and ROS production, resulting in an imbalance in the antioxidant/oxidative ratio (GSH and SOD decrease and MDA increase). As a consequence of the production of inflammatory markers, these events could initiate insulin resistance.

Pioglitazone administration improved biochemical outputs via reduced MDA levels and improved GSH and SOD activities compared to the untreated insulin resistance-induced group. Thus, non-enzymatic and enzymatic antioxidants were decreased in our insulin resistance model. The antioxidant effects of pioglitazone may be beneficial for insulin resistance treatment. Our data was in agreement with Surapaneni and Jainu (31) on the effect of pioglitazone on the oxidant and antioxidant markers.

Both antioxidant and traditional therapeutics reduce fatty liver disease by decreasing oxidative stress. These include N-acetyl-L-cysteine, ursodeoxycholic acid, zinc, vitamin E, and lipoic acid (31). However, such antioxidant and insulin therapeutics require supplementary medication i.e., pioglitazone; its molecular component, PPAR-γ, has a vital role in metabolic homeostasis and controls cellular reactions in atherothrombosis (32). Moreover, pioglitazone significantly decreases TC, ALT, C-reactive protein levels, fasting blood glucose levels, insulin, and HOMA in T2DM (32).

In terms of the impact of HFCD on obesity, inflammatory markers, and T2DM, WAT produces elevated inflammatory cytokines, including TNF-α and IL-6, which exert negative systemic and local effects on WAT and organ functions (9,15). Moreover, WAT is also permeated by macrophages, which are major secretors of pro-inflammatory cytokines. These molecules in combination with HFCD lead to insulin resistance pathogenesis, T2DM, and obesity. Precisely, both IL-6 and TNF-α can modify insulin sensitivity by modulating different important stages in the insulin signaling pathway (12).

Leptin is a signaling molecule derived from adipose tissue, and regulates energy metabolism by inhibiting food intake, generating energy expenditure, and restoring normo-glycemic levels, but leptin resistance limits these functions during obesity (4). Moreover, leptin may modulate TNF-α production and macrophage activation (12). Similarly, resistin is an adipocytokine that is positively correlated with adiposity, potentially connecting obesity with insulin resistance and T2DM (33–35). In a recent study, serum resistin levels were increased in obese mice and the administration of anti-resistin antibodies improved insulin sensitivity (33). Also, serum resistin levels are reportedly raised in obese (34) and diabetic patients (35), but this is controversial (36). Resistin expression is also positively associated with adiposity and insulin resistance (4). In rats, resistin induces insulin resistance, but its effects on insulin sensitivity remain controversial in humans. Our study identified increased leptin and resistin levels in HFCD rats suggesting the presence of insulin resistance.

Insulin resistant conditions, including obesity and T2DM, may lead to chronic low-grade inflammation that can be inverted by pioglitazone therapy together with enhanced insulin action (37).

Bieghs et al. (38) investigated inflammatory reactions in mice fed a high-fat-cholesterol diet for three months, and observed hepatic fibrosis; consequently, it is reasonable to consider that since NAFLD is often linked to obesity and insulin resistance/T2DM, lifestyle adaptation and loss of weight still are the keystones of therapy as they can improve or even reverse the illness.

Unequivocally, changes in glucose and fat metabolism and subsequent hyperglycemia and hypertriglyceridemia contribute to insulin resistance. Chronic constant hyperglycemia and hypertriglyceridemia lead to micro- and macro-vascular complications via oxidative stress (39) and inflammatory biomarkers are detectable with evolving insulin resistance (40). These events have a great importance for biomedical diagnosis and medication therapy programs to fight insulin resistance, which is important factor in obesity, NAFLD, and T2DM disorders.

## Conclusions

The HFCD initiated a metabolic cascade of elevated lipid profiles, hypertriglyceridemia, hyperglycemia, oxidative stress, insulin, and inflammatory biomarkers implicated in insulin resistance and NAFLD. This cascade was broken at several stages of hyperglycemia, hypertriglyceridemia, oxidative stress, inflammation, and insulin resistance using pioglitazone. This drug induces inflammatory reactions in insulin resistance by inhibiting TNF-α and IL-6. Insulin resistance mediated disturbances in antioxidant levels and obesity-associated hormones and inflammatory biomarkers may be targeted by pioglitazone. Moreover, we propose that pioglitazone alleviates HFCD-mediated insulin resistance by stabilizing anti-oxidative and anti-inflammatory factors, and improving insulin sensitivity. Thus, these inflammatory modifications induced by pioglitazone may limit atherothrombotic processes in insulin resistance.
